# Latent Syphilis Infection in Pregnancy: An Ultrasound Diagnosed Case of Penicillin Treatment Failure

**DOI:** 10.1155/2018/8706738

**Published:** 2018-07-26

**Authors:** Lucia Pasquini, Elena Rita Magro-Malosso, Adalgisa Cordisco, Michele Trotta, Mariarosaria Di Tommaso

**Affiliations:** ^1^Maternal and Child Department, Careggi University Hospital Florence, Florence, Italy; ^2^Infectious Disease Unit, Careggi University Hospital Florence, Florence, Italy

## Abstract

We report a case of early latent syphilis (reactive serologic tests without clinical evidence of disease within 24 months from the onset of the infection) in pregnancy. Despite an appropriate maternal treatment with benzathine penicillin G, sonographic signs of fetal syphilis were detected. Follow-up scans, in addiction to serial serological tests, have allowed the identification of fetal infection and therefore the failure of antibiotic therapy. We highlight the importance of ultrasound in suspecting fetal infection and in evaluation of the fetal response after penicillin treatment.

## 1. Introduction

Syphilis in pregnancy continues to be an important public health problem despite the fact that laboratory investigations and effective treatment have been well established for many years. Penicillin is effective against* Treponema pallidum* and represents the drug of choice during pregnancy. However this treatment may not always prevent fetal infection even when administered with a recommended regimen. Treatment failure has also been reported in women treated in the first trimester although the risk of transmission from an infected mother to her fetus seems to be higher in pregnant women treated during the third trimester [1]. We present a case of congenital syphilis in which follow-up scans, in addiction to serial serological tests, have allowed the identification of fetal infection and therefore the failure of antibiotic therapy.

## 2. Case Report

A 29-year-old secundigravida Caucasian woman at 19+5 weeks of gestation was referred to our Fetal Medicine Centre to perform a level II ultrasound scan because of positive serology for syphilis in the first trimester. No maternal clinical manifestations of disease was found on examination. She had had a history of a feverish erythematosus maculopapular rush localized to trunk, limbs, palms, and soles two years before. The diagnosis of syphilis was performed only during antenatal screening in the first trimester by a positive venereal disease research laboratory (VDRL) and a treponema pallidum hemagglutination assay (TPHA) title of 1:2560. The HIV status of the woman was negative. Antibiotic therapy was started immediately with benzathine penicillin G 7.2 million units total, administered as 3 doses of 2.4 million units IM each at 1-week intervals according to the stage of syphilis, in line with CDC guidelines. At the end of the treatment the serology tests were stable with VDRL positive, a TPHA title of 1:2560.

The serology for syphilis also resulted positive in her husband (VDRL positive with TPHA title of 1:320) who was treated with a recommended regimen.

The scan performed at 19+5 weeks of gestation in our centre revealed no abnormalities, the amniotic fluid was found regular, and fetal growth parameters were normal.

**Figure 1:**
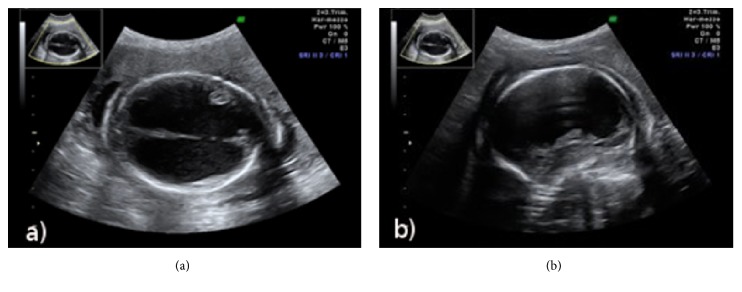
**Hydrocephalus at 23** ^**+5**^ **weeks (a and b)**. 2D transverse (a) and median sagittal section (b) of the fetal brain at 23+5 weeks of gestation. These images show the massive destruction of the cerebral parenchyma and the subversion of the regular intracranial anatomy for the impressive hydrocephalus.

At 23+5 weeks of gestation a follow-up scan was performed and a massive hydrocephalus (Figures 1(a) and 1(b)), severe hydrothorax, ascites, and hepatomegaly were found. The measurement of the middle cerebral artery (MCA) was performed and an increased peak systolic velocity for gestational age was found. Careful counseling with the couple was performed after the ultrasound finding of the fetal anomaly. Maternal blood tests for cytomegalovirus, coxsackievirus, parvovirus, toxoplasma, and herpes virus types 1 and 2 were found negative while syphilis serologic tests were confirmed positive with RPR title 1:4 and TPHA title 1:640. Fetal karyotype was normal. Further counseling was made and the woman decided to pursue the termination of pregnancy. Fetal autopsy showed a cutaneous erythematous papular disseminated rash, widespread edema (stronger in the limbs), ascites, hydrothorax, internal obstructed hydrocephalus, ischemic-haemorrhagic brain injury with disseminated lesions, productive pachymeningitis with dystrophic calcification, hepatomegaly, acute hepatic and splenic hematopoiesis, productive bilateral peripyelitis, pronounced glomerulus-poiesis, and involution of the thymus. The research of Treponema Pallidum with coloring of Warthin-Starry identified images compatible with the morphological appearance of the germ. The diagnostic conclusion deposed for congenital syphilis.

## 3. Conclusions

Maternal syphilis remains an important cause of perinatal morbidity and mortality. Nearly 1.5 million is the estimated global number of pregnancies with probable active syphilis in 2008 [2].

The vertical transmission rate is related to gestational age, the stage of maternal infection during pregnancy, and immunological response of the fetus [3].

Transmission of infection from maternal circulation to the fetus through transplacental transfer of spirochetes can occur as early as 9-10 weeks of gestation although the majority of infections in utero occur in the second trimester [4, 5]. Fetal abnormalities can be detected only after 18-20 weeks, when the fetus starts to become immunocompetent and the dissemination of Treponema in all fetal tissues results in a massive inflammatory response [5].

The risk of vertical transmission is higher in the earlier stages of the disease in the mother probably due to faster replicative capacity of the microorganism and the highest concentration of spirochetes in the blood circulation. Fiumara reported in untreated primary and secondary syphilis a higher risk of transmission (of 70% to 100%) than untreated latent syphilis (40% for the early and up to 10% for the late latent stage) [6].

If left untreated, pregnancies affected by syphilis can be associated with adverse pregnancy outcomes such as premature and low-birth-weight infants, newborns with clinical evidence of syphilis, early fetal loss/stillbirth, and neonatal death [7].

Penicillin remains the treatment of choice during pregnancy for preventing maternal transmission to the fetus and for treating fetal infection [8]. An antibiotic regime depends on the stage of the maternal infection and the HIV status of the mother. For women with early latent infection clinical studies suggest the use of penicillin 2.4 million units IM in a single dose. On the basis of the clinical history obtained and physical examination, our case was an early latent syphilis stage (latency within 24 months) where standard therapy consists of administration of 2.4 million units of penicillin per week for 3 cycles [9].

In a study involving pregnant women with primary, secondary, or early latent syphilis, treatment with a single dose of penicillin prevented fetal infection in 98% of the cases [8].

Patients who have been treated for late latent syphilis showed a low serological response with a persistence of the titers in more than 50 of cases after 5 years [6].

Early diagnosis and treatment are very important factors in the management of syphilis during pregnancy in order to prevent syphilis related adverse pregnancy outcomes. However, in our case, despite the early diagnosis of syphilis in the first trimester of pregnancy, the early latent stage of the syphilis, and the standard antibiotic therapy immediately administered, the obstetric outcome was adverse.

CDC guidelines recommend a sonographic fetal evaluation when syphilis is diagnosed during the second half of pregnancy [9].

We support that all patients who have serological diagnosis of syphilis, at any gestational age, should perform a sonographic fetal evaluation after 18 weeks of gestation. Serial follow-up scans are needed to assess the evolution of the fetal clinical conditions and the response to treatment with penicillin.

In this case report, the first level II ultrasound did not find any fetal abnormality. This may result in an incorrect assumption that the antibiotic therapy had been able to prevent maternal transmission to the fetus. Therefore we believe that a second ultrasound should be repeated after 4 weeks from the first for an appropriate follow-up.

The most common sonographic signs of fetal syphilis (hepatomegaly, ascites, hydrops, fetal anemia, polyhydramnios, and placentomegaly) are associated with a higher risk of treatment failure [10] further with a higher risk of congenital syphilis at delivery [11].

It was found that 12% of neonates with congenital syphilis at birth have a normal antenatal sonographic finding [11]. Nevertheless, in a pregnancy with positive serology for syphilis, an ultrasound scan represents a useful tool in most cases for detecting the main fetal abnormalities caused by congenital infection, and it allows an evaluation of fetal response after penicillin treatment.
